# Editorial: Beneficial Microbes and the Interconnection Between Crop Mineral Nutrition and Induced Systemic Resistance

**DOI:** 10.3389/fpls.2021.790616

**Published:** 2021-11-29

**Authors:** Carlos Lucena, Sabine Dagmar Zimmermann, Jianfei Wang, Ricardo Aroca

**Affiliations:** ^1^Departamento de Agronomía (DAUCO-María de Maeztu Unit of Excellence), Universidad de Córdoba, Córdoba, Spain; ^2^BPMP, CNRS, INRAE, Institut Agro, Université de Montpellier, Montpellier, France; ^3^Anhui University of Science and Technology, Huainan, China; ^4^Departamento de Microbiología del Suelo y Sistemas Simbióticos, Estación Experimental del Zaidín (CSIC), Granada, Spain

**Keywords:** nutrient deficiency, ISR eliciting microbes, crops, soil, microbial consortia

To cope with nutrient deficiencies, plants develop morphological and physiological responses, mainly in their roots, aimed to facilitate nutrient acquisition (Lucena et al., 2018). In the last years, it has been found that some rhizosphere microbes can induce physiological and morphological responses in roots of dicot plants similar to the ones induced by plants under nutrient deficiencies (Verbon et al., 2017). Remarkably, these rhizosphere microbes are also capable of eliciting the induced systemic resistance (ISR) against pathogens and insects (Pieterse et al., 2014; Verbon et al., 2017). This observation suggests that both processes (ISR and nutrient deficiency responses) are closely interconnected thus opening new possibilities for optimizing the management of the rhizosphere microbiota for improving mineral nutrition and health (Zamioudis et al., 2015; Verbon et al., 2017, 2019). However, the nodes of convergence between the two processes remain unclear (Romera et al., 2019). Elucidating the main nodes of interconnection between the pathways regulating microbe-elicited ISR and mineral uptake is critical for optimizing the use of plant mutualistic microbes in agriculture. The Research Topic updates latest findings related to the roles of ISR eliciting microbes in crops. It includes 12 original articles and one review, eight articles are related to beneficial microbes as biocontrol effectors inducing disease resistance and growth promotion of their hosts (Cueva-Yesquén et al.; La Spada et al.; Qu et al.; Qin et al.; Tseng et al.; Yu et al.; Zhou et al.; Zhu et al.), two articles concern growth promotion under abiotic stress (Tseng et al.; Yuan et al.), two others are linked to reduced chemical fertilization or soil property changes (Cardoso et al.; Wang et al.), and one to the role of N_2_ fixing bacteria (Kordi et al.). The review is related to the special role of mycorrhizal fungi on orchid seed germination (Zhao et al.).

Regarding the role of diverse microorganisms as biocontrol effectors, a variability of physiological and molecular mechanisms has been observed. The tight link between beneficial effects of microorganisms on plant growth by improved nutrition and defense priming through systematic enhancement of resistance against below-ground and above pathogens or insect herbivores has been described for arbuscular mycorrhizal (AM) fungi as mycorrhiza-induced resistance (MIR) (Cameron et al., 2013). Such interactions might be dependent on abiotic factors and mediated by jasmonic acid (JA) signaling. This interconnection between *Plantago lanceolate* with the AM fungus *Funneliformis mosseae* and the herbivore *Mamestra brassica* was studied by Qu et al.. Surprisingly, in contrast to tomato (Rivero et al., 2021), they reported in their specific case a repression of JA-mediated defense by AM fungi, underlining the complexity of the studied model under their selected conditions (symbiotic and pathogen partners, age of plants, light, soil P, JA treatments). Mycoparasitic *Trichoderma* fungi have been used as biocontrol agents (Guzmán-Guzmán et al., 2019). La Spada et al. demonstrated that two selected *Trichoderma* strains (*T. asperellum* and *T. atroviride*) promoted tomato growth and reduced the disease severity caused by the oomycete *Phytophthora nicotianae*. Genetic patterns of the components of the experimental model tomato–*Trichoderma* spp.–*P. nicotianae* were differentially modified. Both counteract the challenge of infections by modulating the expression of crinkler, necrosis-inducing *Phytophthora* protein 1, and cellulose-binding elicitor lectin pathogenic effectors involved in plant defense mechanisms. Tseng et al. isolated a new endophytic fungus (a *Trichoderma* strain) from the leaves of a deciduous wood tree *Leucas aspera*. When applied to *Arabidopsis thaliana* and *Nicotiana attenuata*, this fungus colonized their roots thereby strongly promoting the initial plant growth in soil. The fungus showed predatory capability on the pathogenic fungus *Alternaria brassicicola*. Colonized *A. thaliana* plants displayed lower *A. brassicicola* spread in roots and shoots, while AM formation in *N. attenuata* was not affected by the *Trichoderma* strain.

Plant growth promoting bacteria (PGPB) living as endophytes display several beneficial traits as improving nutrient bioavailability, interfering with hormone levels, or protection against abiotic and biotic stress (de Souza et al., 2015; Kumar et al., 2020) thus leading also to better plant growth, development, and resistance. Cueva-Yesquén et al. isolated and analyzed such culturable bacterial endophytes from passionflower (*Passiflora incarnata*) by phenotypic and genotypic approaches and confirmed finally the probiotic effect of some of them by evaluating their capacity to boost germination and growth of another plant, namely of the Cape gooseberry (*Physalis peruviana*). In another study, in the context of rhizobacteria-mediated defense, Zhu et al. reported that the PGPB *Pseudomonas fluorescens* could increase the resistance of cucumber plants against infection by *Botrytis cinerea*. *Pseudomonas* bacteria have been used before as biocontrol effectors in different plant species (Kupferschmied et al., 2013; De Vrieze et al., 2020) and different mechanisms were proposed. Here, the authors found by RNA-sequencing that the improved defense would be linked to the expression of polyamine-associated and defense-related genes. Zhou et al. demonstrated the ability of the rhizobacterial strain *Bacillus subtilis* SL18r to trigger ISR in tomato plants against the foliar pathogen *Botrytis cinerea*. The authors reported that the long non-coding RNAs (lncRNAs) were involved in the mediation of the rhizobacteria-primed ISR processes in plants by a comparative transcriptome analysis between non-inoculated and SL18r-inoculated plants. Postharvest strawberry is susceptible to gray mold disease caused by *B. cinerea*. Yu et al. found that inoculation with *Bacillus cereus* diminished disease severity by modulating salicylic acid (SA) pathway as revealed by transcriptomic analysis, and enhancing antioxidant activity of strawberry fruits. Finally, the cotton seedling response to *Bacillus circulans* GN03 was explored by Qin et al. showing a remarkably enhanced growth promotion as well as disease resistance. GN03 inoculation altered the microbiota in and around the plant roots. At the physiological and molecular level, the authors observed a significant accumulation of growth-related (indole acetic acid (IAA), gibberellic acid (GA), and brassinosteroids) and disease resistance-related hormones (SA, JA), an up-regulated expression of phytohormone synthesis-related genes (EDS1, AOC1, BES1, GA20ox), of an auxin transporter gene (Aux1), and of disease-resistance genes (NPR1, PR1).

Regarding abiotic stress, it is well-known that PGPB enhance salt tolerance of plants by several mechanisms (Kumar et al., 2020), one of them might be the production of some organic compounds. One of these compounds are phenazines, a class of diffusible, heterocyclic compounds harboring substitutions of various functional groups on the core of the phenazine ring structure. Thus, Yuan et al. demonstrated with *Pseudomonas chlororaphis* defective or overproducing strains, that phenazine production improved the efficiency in increasing wheat salt tolerance. The fungal *Trichoderma* strain isolated by Tseng et al. acting as biocontrol effector (see above) could grow on high NaCl or mannitol concentrations and improved salt tolerance of colonized *A. thaliana*.

With respect to soil nutrient conditions, results obtained by Cardoso et al. showed the potential ability of the PGPB strain *Bacillus cereus* UFRABC40 to promote the growth performance of coconut seedlings under decreased application of inorganic fertilizers. Seedling treatments by 100% chemical fertilizer NPK or 50% NPK together with *B. cereus* indicated that the inoculation increased phytohormone levels (IAA, GA) and leaf gas exchange (by assimilation of CO_2_, stomatal conductance to water vapor, transpiration and instantaneous carboxylation efficiency). Furthermore, growth parameters and macro- and micronutrient levels were improved. More generally, Wang et al. found a change of bacterial diversity and community together with soil properties and plant functioning during long-term grassland restoration and recovery. The observed changes in soil microbial community were tightly linked to the presence of increased soil C and N substrates due to plant growth and diversity. However, whether these bacterial changes improved growth and tolerance of plants to facilitate the recovery of the analyzed grassland ecosystem remains to be further studied.

Finally, the use of N_2_ fixing bacteria in the field to increase essential oil (EO) quantity and quality of sweet basil was explored by Kordi et al.. These authors found that application of free living N_2_ fixing bacteria *Azospirillum brasilense* and *Azotobacter chroococcum* together with 50% of regular chemical fertilizer enhanced EO quantity and quality, more than when intercropped with maize plants.

The review concerns orchids being among the most endangered in the plant kingdom. Lack of endosperm in their seeds renders orchids to depend on nutrients provided by orchid mycorrhizal fungi (OMF) for seed germination and seedling formation in the wild (Li et al., 2021). Zhao et al. presented a new technology using seed germination-promoting OMF coming from roots or seeds of orchid plants to be used for reintroduction of orchids in their natural habitat.

In conclusion, this Research Topic, by putting together different beneficial and nutritional aspects affected by a diversity of microorganisms, tries to pave the way for future research about their role in plant mineral nutrition linked to ISR aiming finally in their better use and involvement in a more sustainable and environmentally friendly agriculture.

## Author Contributions

RA, SZ, and CL reviewed and summarized the articles of the Topic. CL wrote the draft. All authors revised and validated the final manuscript.

## Conflict of Interest

The authors declare that the research was conducted in the absence of any commercial or financial relationships that could be construed as a potential conflict of interest.

## Publisher's Note

All claims expressed in this article are solely those of the authors and do not necessarily represent those of their affiliated organizations, or those of the publisher, the editors and the reviewers. Any product that may be evaluated in this article, or claim that may be made by its manufacturer, is not guaranteed or endorsed by the publisher.
